# Intracholecystic papillary neoplasm acquiring malignant characteristics and leading to multiple liver metastases: A case report

**DOI:** 10.1002/jgh3.12994

**Published:** 2023-11-10

**Authors:** Tsuyoshi Suda, Yasunori Sato, Yusuke Ito, Kiichiro Kaji, Shuichi Terasaki, Yasuni Nakanuma

**Affiliations:** ^1^ Department of Gastroenterology Kanazawa Red Cross Hospital Kanazawa Japan; ^2^ Department of Human Pathology Kanazawa University Graduate School of Medicine Kanazawa Japan; ^3^ Department of Diagnostic Pathology Shizuoka Cancer Center Shizuoka Japan; ^4^ Department of Diagnostic Pathology Fukui Prefecture Saiseikai Hospital Fukui Japan

**Keywords:** adenocarcinoma, biomarkers, case reports, gallbladder neoplasms, genes, p53, papillary, tumor

## Abstract

The mechanisms underlying the progression of intracholecystic papillary neoplasms (ICPNs) to gallbladder cancer and invasive cancer remain relatively unclear. In the present case, metastatic liver tumors were suspected in an 83‐year‐old man at presentation; however, the primary tumor was unknown. The patient died shortly thereafter as a result of rapid tumor progression. An autopsy revealed multiple liver, lung, and lymph node metastases. Additionally, a fragile papillary tumor with a high‐grade dysplastic epithelium with tubulopapillary morphology and admixed foci of a low‐grade dysplastic epithelium were detected at the fundus of the gallbladder. The well‐differentiated tubular adenocarcinoma had extensively invaded the wall's granular mucosal surface along with the solitary papillary tumor. Based on pathological findings, a diagnosis of an ICPN with an associated invasive carcinoma was established. This case is novel because it showed that an ICPN can progress aggressively.

## Graphical abstract

We report a case of invasive gallbladder cancer with multiple liver metastases derived from the intracholecystic papillary neoplasm (ICPN). Progression of invasive gallbladder cancer from ICPN could be followed pathologically. This case suggests that unresectable gallbladder cancers of ICPN origin may remain overlooked, potentially resulting in poor prognosis.

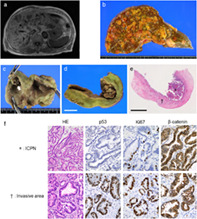

## Introduction

According to the World Health Organization classification (fifth edition), an intracholecystic papillary neoplasm (ICPN) is a noninvasive, intraepithelial tumor presenting as a polypoid papillary mass arising from the gallbladder.^1^ Approximately 6.4% of all gallbladder neoplasms are associated with ICPNs. Compared with patients who have conventional gallbladder neoplasms, those with ICPNs generally have a better prognosis.^2^ However, the development of invasive gallbladder cancer from ICPNs remains inadequately documented.

We report a case of invasive gallbladder cancer (originated from an ICPN) and multiple liver metastases, which were pathologically examined in detail.

## Case report

**Figure 1:**
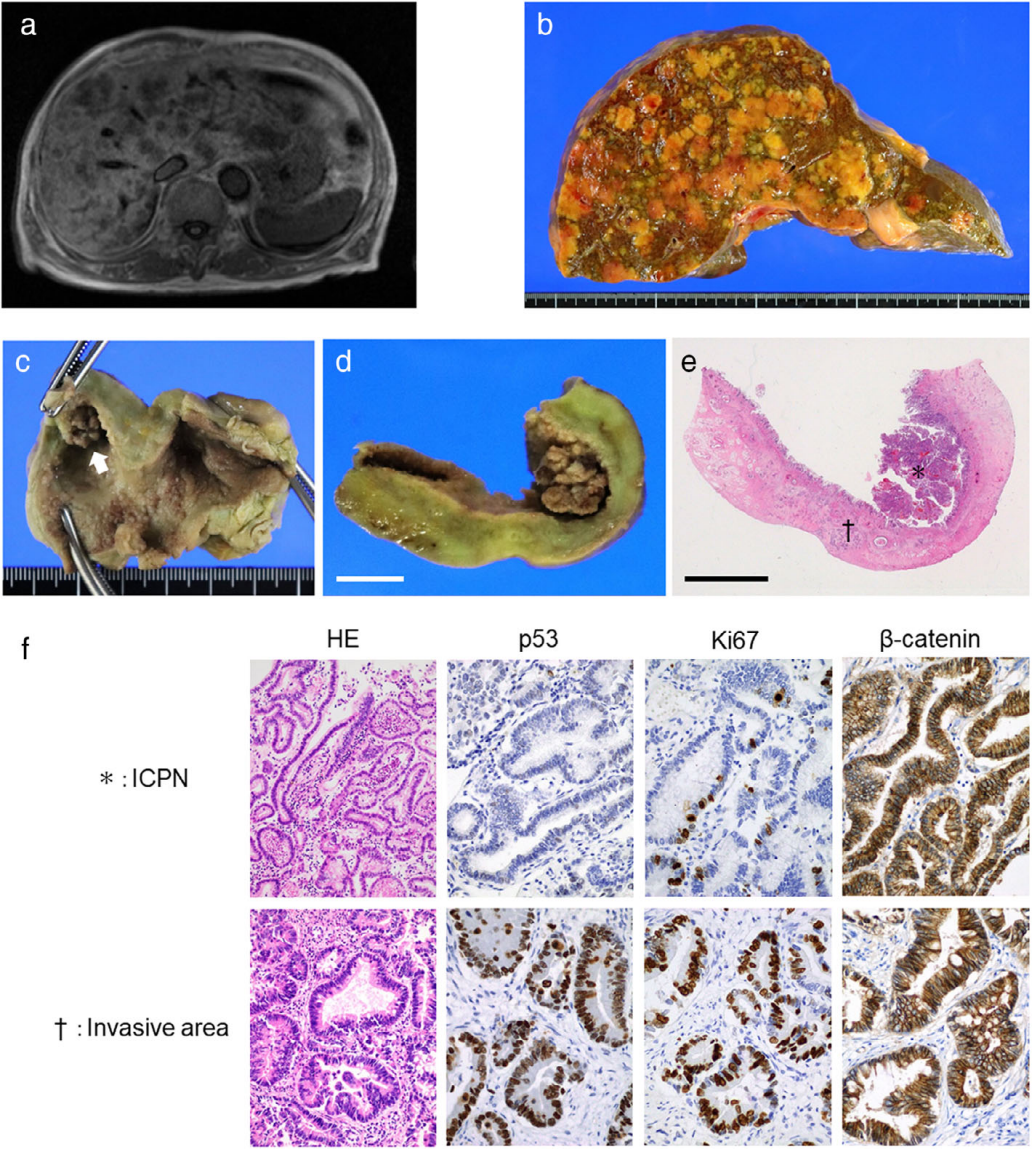
Diagnostic findings of the liver tumors and the gallbladder tumor. (a) Multiple liver tumors as seen on magnetic resonance imaging. (b) Cut surface of the liver showing multiple metastases. (c) Gross appearance of the gallbladder (after formalin fixation). The arrow indicates a papillary tumor at the fundus. (d) Cut surface of the gallbladder showing a papillary tumor and thickening of the gallbladder wall. Bar, 1 cm. (e) Whole‐mount view of the gallbladder stained with hematoxylin and eosin (H&E). (*) and (†) denote an intracholecystic papillary neoplasm and the area of the invasive carcinoma, respectively. (f) Histologic findings of the gallbladder tumor. Representative images of H&E staining and immunostaining for p53, Ki‐67, and β‐catenin are shown. Original magnifications: H&E, ×200; immunostaining, ×400.

An 83‐year‐old male patient incidentally presented with multiple liver metastases (Fig. 1a). Tumor markers, namely carcinoembryonic antigen (CEA; 102.6 ng/mL) and carbohydrate antigen (CA) 19–9 (58.2 U/mL), were found to be elevated. Although metastatic liver tumors were suspected, the primary tumor was unknown. The patient died shortly after presentation because of rapid tumor progression.

An autopsy revealed multiple liver metastases (Fig. 1b) along with lung and lymph node metastases. A fragile papillary tumor measuring 1 cm was detected at the fundus of the gallbladder (Fig. 1c [arrow], d). Additionally, the fundus wall and gallbladder body were diffusely thickened, with the surface mucosa exhibiting a flat but fine granular appearance.

Histologically, the papillary tumor on the gallbladder fundus was mainly composed of a high‐grade dysplastic epithelium with a tubulopapillary morphology, corresponding to a well‐differentiated adenocarcinoma (Fig. 1e,f). The foci of a low‐grade dysplastic epithelium were also admixed. Within the thickened walls of the gallbladder fundus and body, the well‐differentiated tubular adenocarcinoma had extensively invaded the wall's granular mucosal surface (Fig. 1e,f) along with the solitary papillary tumor. Based on these pathological findings, a diagnosis of an ICPN with an associated invasive carcinoma was established.

Immunostaining revealed that the ICPN and invasive carcinoma were positive for cytokeratin (CK)7, CEA, CA19‐9, mucin (MUC)1, and MUC5AC. Similar results were obtained following immunostaining of the liver metastases.

Immunostaining further revealed that the ICPN was negative for tumor protein p53 (Fig. 1f); however, the invasive carcinoma of the gallbladder was diffusely positive for p53. This indicated that the carcinoma, and not the ICPN, harbored a *TP53* mutation. The Ki‐67 expression, which is a proliferation marker, was more elevated in the invasive carcinoma than in the ICPN (Ki‐67‐labeling indices: 71% *vs* 10%). Diffuse and intense β‐catenin immunoreactivities were observed in the cytoplasm and cellular membranes of the cells from both the ICPN and invasive carcinoma. Similarly, both lesions lacked nuclear β‐catenin expression (Fig. 1f). Immunostaining of the liver metastases for p53, Ki‐67, and β‐catenin yielded almost identical findings as those of the invasive carcinoma of the gallbladder.

## Discussion

ICPNs reportedly have a good prognosis^2^; however, previous studies have evaluated only resectable cases.^2^, ^3^, ^4^ In a recent retrospective study, T‐stage matching was performed to account for the higher prevalence of T1 or lower stages in patients with ICPN compared to that of those with conventional gallbladder cancer. After T‐stage matching, the result revealed no differences in prognosis between ICPNs and conventional gallbladder cancer.^5^ Therefore, it remains unclear whether ICPNs are indeed a group of diseases with a good prognosis.

Invasion of ICPNs into the stroma has been reported to be associated with irregular histologic findings, complicated lesions, diffuse high‐grade dysplasia, tubulopapillary/papillary growth patterns, a large tumor size, and a high Ki‐67 proliferation index.^3^, ^4^ However, additional yet‐to‐be‐identified factors may also play a role in ICPN invasion. In our case, no *TP53* mutation was present in the ICPN initially; the mutation was believed to have developed during proliferation, resulting in invasion and metastasis.

p53 is considered to be associated with the early stages of tumorigenesis in gallbladder tumors.^6^ Moreover, our results were consistent with those of a previous study which revealed a high incidence of abnormal p53 expression in the invasive components of ICPN‐associated gallbladder cancer.^7^

Furthermore, unlike typical gallbladder cancer, ICPNs are known to have an altered Wnt/β‐catenin signaling pathway.^8^ β‐Catenin overexpression was also observed in our case, thereby complementing the diagnosis of ICPN‐derived gallbladder cancer.

To our knowledge, there are no previous reports on ICPNs accompanied by liver metastases and a poor prognosis. We believe that our case may be representative of several unresectable gallbladder cancers of an ICPN origin that may be overlooked because of their poor prognoses.

Ethics Approval: The identity of the patient has been protected.

Patient Consent: Informed consent was obtained from the deceased patient's family for this report.

## References

[1] Nagtegaal ID , Odze RD , Klimstra D *et al*. The 2019 WHO classification of tumours of the digestive system. Histopathology. 2020; 76: 182–188. 31433515 10.1111/his.13975 PMC7003895

[2] Adsay V , Jang KT , Roa JC *et al*. Intracholecystic papillary‐tubular neoplasms (ICPN) of the gallbladder (neoplastic polyps, adenomas, and papillary neoplasms that are ≥1.0 cm): clinicopathologic and immunohistochemical analysis of 123 cases. Am. J. Surg. Pathol. 2012; 36: 1279–1301. 22895264 10.1097/PAS.0b013e318262787c

[3] Nakanuma Y , Nomura Y , Watanabe H *et al*. Pathological characterization of intracholecystic papillary neoplasm: a recently proposed preinvasive neoplasm of gallbladder. Ann. Diagn. Pathol. 2021; 52: 151723. 33725666 10.1016/j.anndiagpath.2021.151723

[4] Argon A , Barbet FY , Nart D . The relationship between intracholecystic papillary‐tubular neoplasms and invasive carcinoma of the gallbladder. Int. J. Surg. Pathol. 2016; 24: 504–511. 27122163 10.1177/1066896916644781

[5] Kang JS , Lee KB , Choi YJ *et al*. A comparison of outcomes in patients with intracholecystic papillary neoplasms or conventional adenocarcinomas of the gallbladder. HPB (Oxford). 2021; 23: 746–752. 33092965 10.1016/j.hpb.2020.09.011

[6] Wistuba II , Gazdar AF , Roa I , Albores‐Saavedra J . p53 protein overexpression in gallbladder carcinoma and its precursor lesions: an immunohistochemical study. Hum. Pathol. 1996; 27: 360–365. 8617479 10.1016/s0046-8177(96)90109-4

[7] Mochidome N , Koga Y , Ohishi Y *et al*. Prognostic implications of the coexisting precursor lesion types in invasive gallbladder cancer. Hum. Pathol. 2021; 114: 44–53. 33989638 10.1016/j.humpath.2021.05.001

[8] Akita M , Fujikura K , Ajiki T *et al*. Intracholecystic papillary neoplasms are distinct from papillary gallbladder cancers: a clinicopathologic and exome‐sequencing study. Am. J. Surg. Pathol. 2019; 43: 783–791. 30807303 10.1097/PAS.0000000000001237

